# Risk factors for bowel necrosis in patients with hepatic portal venous gas

**DOI:** 10.1007/s00595-014-0941-1

**Published:** 2014-06-01

**Authors:** Hiroyuki Koami, Tsutomu Isa, Tomonari Ishimine, Shinichiro Kameyama, Toshinobu Matsumura, Kosuke Chris Yamada, Yuichiro Sakamoto

**Affiliations:** 1 Department of Surgery, Center for Gastroenterology, Urasoe General Hospital, Iso 4-16-1, Urasoe, 901-2132 Japan; 2 Department of Emergency and Critical Care Medicine, Faculty of Medicine, Saga University, Nabeshima 5-1-1, Saga, 849-8501 Japan

**Keywords:** Hepatic portal venous gas, Bowel necrosis, MDCT, Logistic regression analysis, Diagnostic criteria

## Abstract

**Purpose:**

To evaluate the risk factors for bowel necrosis in adult patients with hepatic portal venous gas (HPVG).

**Methods:**

This retrospective study comprised 33 adult patients treated for HPVG between August, 2008 and December, 2011. The patients were divided into a necrotic group (*n* = 14) and a non-necrotic group (*n* = 19). We analyzed the clinical demographics, laboratory data, multi-detector computed tomography findings, treatments, and outcomes in each group.

**Results:**

Abdominal pain, peritoneal signs, systolic blood pressure, aspartate aminotransferase, alanine aminotransferase, lactate dehydrogenase (LDH), small intestinal dilatation, poor enhancement of the bowel wall, and intestinal pneumatosis were all significantly associated with bowel necrosis. Moreover, there were significantly more operative cases and deaths in the necrotic group. Multivariate analysis revealed that systolic BP (*p* = 0.048), LDH (*p* = 0.022), and intestinal pneumatosis (*p* = 0.038) were independent risk factors for bowel necrosis. Thus, we created new diagnostic criteria for bowel necrosis based on these three factors, the sensitivity, specificity, and accuracy of which were 100, 78.9, and 87.9 %, respectively.

**Conclusions:**

This study demonstrates new and important findings to evaluate the risk factors for bowel necrosis. Using our diagnostic criteria, the indications for emergency laparotomy can be established more accurately.

## Introduction

Hepatic portal venous gas (HPVG) was initially described in 1955, in neonates with necrotizing enterocolitis [1]. In 1978, Liebman et al. [2] reported that gas in the portal vein was associated with a mortality rate of 75 %. For half a century, HPVG has been considered a poor prognostic factor and an absolute indication for emergency laparotomy [3]. However, a cumulative review of 182 cases of HPVG in adults revealed 38 % mortality in those treated surgically and 39 % in those treated conservatively, without a significant difference in mortality between the groups [4]. In recent years, there have been many case reports of milder disease courses. Faberman et al. [5] reported a mortality rate of only 29 % in 17 patients with portal venous gas seen on computed tomography (CT) and pointed out that HPVG is itself not a predictor of mortality. However, few studies have reported the relationship between HPVG and disease severity. The purpose of our study was to demonstrate the risk factors for bowel necrosis in patients with HPVG.

## Materials and methods

### Patient data

**Fig. 1:**
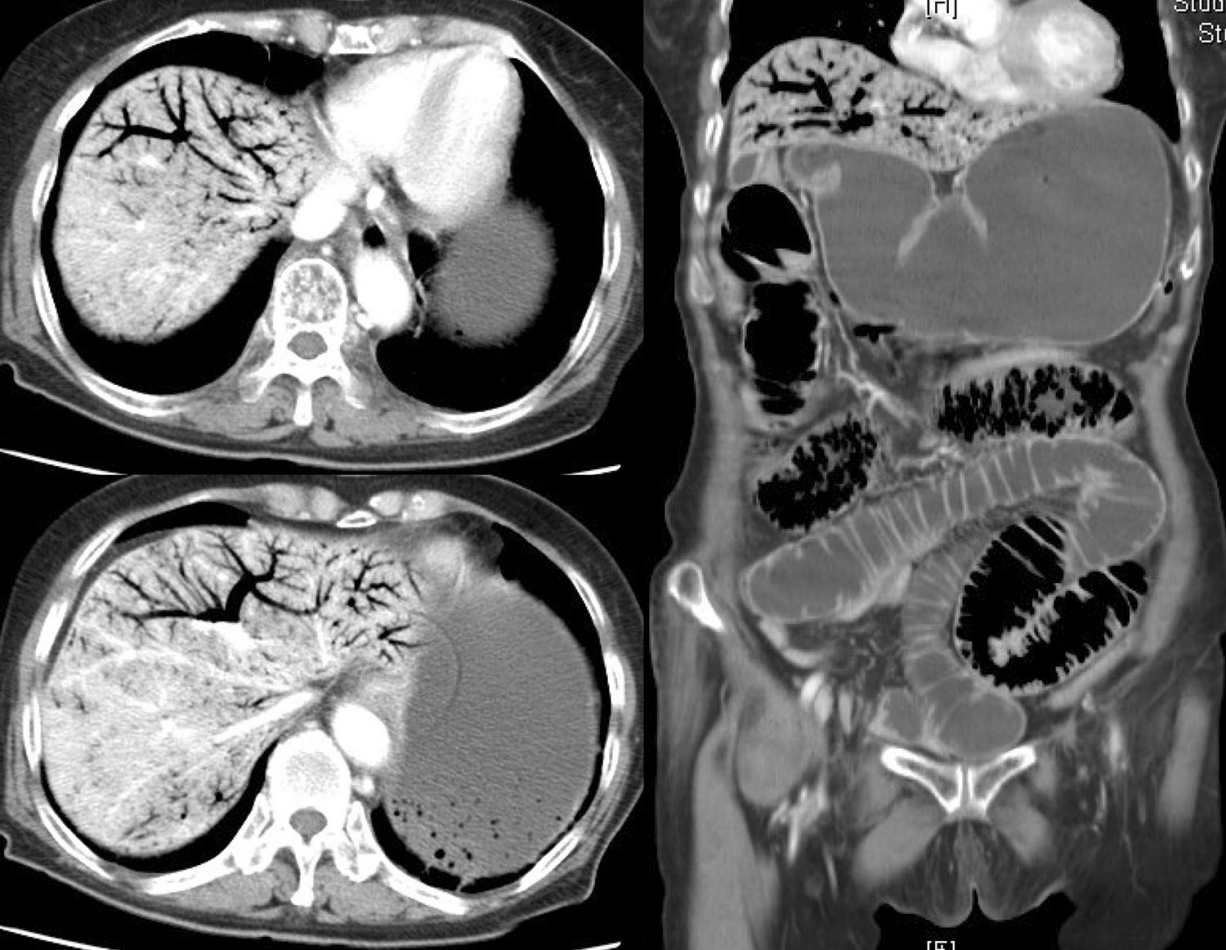
Multi-detector computed tomography (MDCT) findings of the patients with hepatic portal venous gas (HPVG)

**Fig. 2:**
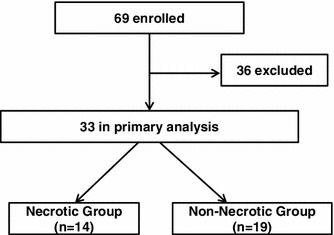
Study design. Thirty-three patients were divided into two groups based on the presence of bowel necrosis/ischemia

This retrospective study included all abdominal multi-detector CT (MDCT) scans obtained at one institution, Urasoe General Hospital, between August, 2008 and December, 2011 (Fig. 1). We reviewed the data of 69 patients with HPVG evident on MDCT, retrieved from a computer search. Thirty-six of the 69 patients were excluded from this study because their scans were performed to detect the causes of cardiopulmonary arrest. The remaining 33 patients were divided into two groups based on the presence of bowel necrosis or ischemia: a necrotic group (*n* = 14) and a non-necrotic group (*n* = 19; Fig. 2). We established the presence of bowel necrosis according to the pathological reports and surgical findings. On the other hand, in patients who did not undergo surgery, the bowel necrosis was diagnosed based on the interpretation of radiologists, as we described previously. We analyzed the clinical demographics, including age, sex, admission, abdominal pain, vomiting, peritoneal signs, shock, systolic blood pressure (BP), heart rate, body temperature, and respiratory rate; laboratory data, including white blood cell count (WBC), c-reactive protein (CRP), pH, base excess (BE), total-bilirubin (T-Bil), aspartate aminotransferase (AST), alanine aminotransferase (ALT), creatine kinase (CK), and lactate dehydrogenase (LDH); MDCT findings, including ascites, free air, gastroduodenal dilatation, small intestinal dilatation, large intestinal dilatation, poor enhancement of the bowel wall, intestinal pneumatosis, mesenteric pneumatosis, and gas in the portal vein; diagnoses; treatments; and outcomes of the patients in each group.

### Statistical analysis

To compare differences between the necrotic and non-necrotic groups, Student’s *t* test, the Mann–Whitney *U* test, the Chi square test, or Fisher’s exact test were used as applicable. The factors with significant differences in the univariate analysis were evaluated in a multivariate analysis. We selected the logistic regression analysis (Forward: LR method) for multivariate analysis. Cut-off values were calculated using the factors with significant differences in the multivariate analysis and used to create diagnostic criteria for bowel necrosis. The data were statistically analyzed using SPSS for Windows version 19. Data are expressed as the number of patients and ratios (%) or mean ± standard deviation (or median ± quartile deviation). Values of *p* < 0.05 were considered significant.

## Results

This study comprised 19 men and 14 women, with a mean age of 76 years (range 51–93 years). Of the 33 patients, 10 had bowel obstruction, 7 had non-occlusive mesenteric ischemia, 4 had ischemic colitis, 3 had supra-mesenteric artery thrombosis, 1 had liver injury, and 8 had other diseases or complications.

### Univariate and multivariate analyses

According to univariate analysis, age and male gender distribution was not significantly different in the two groups (Table 1). Among the various parameters examined, abdominal pain (*p* = 0.006), peritoneal signs (*p* = 0.036), systolic BP (*p* = 0.047), AST (*p* = 0.012), ALT (*p* = 0.038), LDH (*p* = 0.019), small intestinal dilatation (*p* = 0.030), poor enhancement of the bowel wall (*p* = 0.012), and intestinal pneumatosis (*p* = 0.030) were each found to be associated with bowel necrosis (Table 1). There were significantly more operative cases (*p* = 0.017) and deaths (*p* = 0.049) in the necrotic group. All four patients who survived in the necrotic group underwent surgery. Multivariate analysis revealed that systolic BP [odds ratio (OR) 0.964, 95 % confidence interval (CI) 0.929–1.000, *p* = 0.048], LDH (OR 1.007, 95 % CI 1.001–1.014, *p* = 0.022), and intestinal pneumatosis (OR 37.793, 95 % CI 1.229–1162.062, *p* = 0.038) were independent risk factors for bowel necrosis (Table 2).

**Table 1:** Baseline clinical characteristics

	Necrotic group (*n* = 14)	Non-necrotic group (*n* = 19)	*P* value (*p* < 0.05)
Clinical demographics			
Age	73.8 ± 9.2	77.4 ± 12.1	0.355^†^
Male	8 (57.1 %)	11 (57.9 %)	0.966
Admission	9 (64.3 %)	8 (42.1 %)	0.208
Abdominal pain	9/10 (90.0 %)	4/13 (30.8 %)	0.006
Vomit	8/13 (61.5 %)	8 (42.1 %)	0.280
Peritoneal signs	7/13 (53.8 %)	3/18 (16.7 %)	0.036
Shock	8 (57.1 %)	5 (26.3 %)	0.073
Systolic BP (mmHg)	91.5 ± 30.3	112.4 ± 27.3	0.047^†^
HR (/min)	117.5 ± 30.0	103.0 ± 32.0	0.161^‡^
BT (°C)	36.8 ± 1.4	36.8 ± 1.1	0.947^†^
RR (/min)	24.5 ± 9.3	24.0 ± 10.0	0.442^‡^
Laboratory data			
WBC (/μL)	15150 ± 12050	13200 ± 9400	0.122^‡^
CRP (mg/dL)	12.7 ± 10.8	7.6 ± 12.2	0.110^‡^
pH	7.37 ± 0.14	7.40 ± 0.21	0.781^‡^
BE	−4.2 ± 8.10	−1.3 ± 10.18	0.430^†^
T-BiL (mg/dL)	0.7 ± 1.0	0.6 ± 0.6	0.567^‡^
AST (U/L)	82.5 ± 289.0	31.0 ± 24.0	0.012^‡^
ALT (U/L)	52.5 ± 68.0	20.0 ± 22.0	0.038^‡^
CK (U/L)	81.0 ± 833.5	65.0 ± 56.5	0.286^‡^
LDH (U/L)	454.5 ± 469.5	232.0 ± 115.0	0.019^‡^
MDCT findings			
Ascites	8 (57.1 %)	11 (57.9 %)	0.622
Free air	0 (0.0 %)	2 (10.5 %)	0.324
Gastroduodenal dilatation	11 (78.6 %)	10 (52.6 %)	0.126
Small intestinal dilatation	13 (92.9 %)	11 (57.9 %)	0.030
Large intestinal dilatation	9 (64.3 %)	11 (57.9 %)	0.710
Poor enhancement of the bowel wall	7/9 (77.8 %)	2/11 (18.2 %)	0.012
Intestinal pneumatosis	13 (92.9 %)	11 (57.9 %)	0.030
Mesenteric pneumatosis	11 (78.6 %)	9 (47.4 %)	0.070
Gas in the portal vein	10 (71.4 %)	8 (42.1 %)	0.095
Treatment and outcome			
Operation performed	8 (57.1 %)	3 (15.8 %)	0.017
Dead	10 (71.4 %)	7 (36.8 %)	0.049

*BP* Blood pressure, *HR* heart rate, *BT* body temperature, *RR* respiratory rate, *WBC* white blood cell, *CRP* c-reactive protein, *BE* base excess, *T-Bil* total-bilirubin, *AST* aspartate aminotransferase, *ALT* alanine aminotransferase, *CK* creatine kinase, *LDH* lactate dehydrogenase, *MDCT* multi-detector CT

^†^ Mean ± standard deviation (*t* test)

^‡^ Median ± interquartile range (Mann–Whitney test)

**Table 2:** Logistic regression analysis for predicting bowel necrosis

	Partial regression coefficient	*P* value	Odds ratio	95 % CI
Systolic BP	−0.037	0.048	0.964	(0.929–1.000)
LDH	0.007	0.022	1.007	(1.001–1.014)
Intestinal pneumatosis	3.632	0.038	37.793	(1.229–1162.062)
Constant	−1.906	0.461		

*CI* confidence interval, *BP* blood pressure, *LDH* lactate dehydrogenase

### Calculating the cut-off values and creating the criteria

**Fig. 3:**
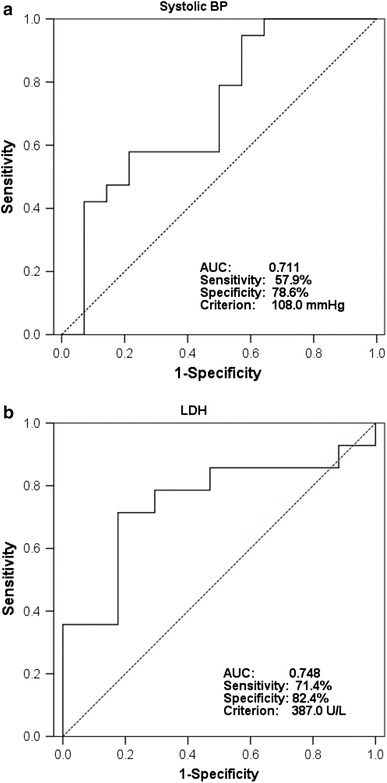
Receiver-operating characteristic (ROC) curves for detecting bowel necrosis according to systolic blood pressure (BP) (**a**) and lactate dehydrogenase (LDH) (**b**). *ROC* receiver-operating characteristic, *AUC* area under the curve, *BP* blood pressure, *LDH* lactate dehydrogenase

**Fig. 4:**
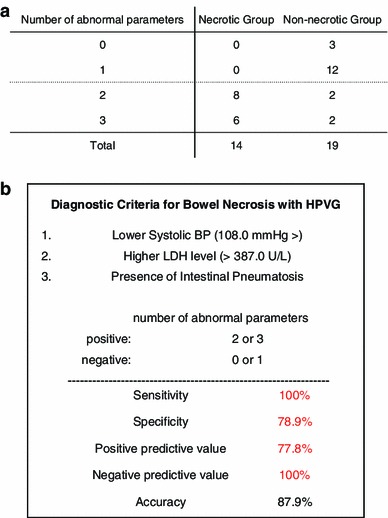
Number of abnormal parameters in the two groups (**a**). New diagnostic criteria for bowel necrosis in the patients with hepatic portal venous gas (HPVG) (**b**). According to our criteria, positive bowel necrosis was defined by more than two abnormal findings. The sensitivity was 100 %, the specificity was 78.9 % and the accuracy was 87.9 %. *HPVG* hepatic portal venous gas, *BP* blood pressure, *LDH* lactate dehydrogenase

In the receiver-operating characteristic (ROC) curve analysis of bowel necrosis, the cut-off value of systolic blood pressure was 108.0 mmHg, the area under the curve (AUC) was 0.711, the sensitivity was 57.9 %, and the specificity was 78.6 % (Fig. 3a). Furthermore, the cut-off value of LDH was 387.0 U/L, the AUC was 0.748, the sensitivity was 71.4 %, and the specificity was 82.4 % (Fig. 3b). The sensitivity of the presence of intestinal pneumatosis was 54.2 % and the specificity was 88.9 %. Next, we examined the number of each of the three factors indicated in the abnormal findings for each patient. All patients in the necrotic group had two or more abnormalities (Fig. 4a). We created diagnostic criteria for bowel necrosis based on three factors; namely, lower systolic BP (108.0 mmHg>), higher LDH level (>387.0 U/L), and the presence of intestinal pneumatosis (Fig. 4b). Based on our criteria, bowel necrosis was diagnosed when a patient had more than two abnormal factors. Importantly, our criteria detected necrotic bowel with a sensitivity of 100 %, a specificity of 78.9 % and an accuracy of 87.9 %.

## Discussion

In this study, we identified the risk factors for bowel necrosis in patients with HPVG and created new diagnostic criteria with high sensitivity and accuracy. These criteria consist of three factors that can be easily assessed by physicians in the emergency department and help establish whether unstable patients who complain of acute abdominal pain have bowel necrosis.

The number of cases of HPVG treated conservatively has been increasing rapidly; however, few reports have addressed the factors that indicate bowel necrosis and no consensus has been reached. MDCT has become the first choice for HPVG detection and evaluation of the underlying process [6]. CT scans are more sensitive than plain radiographs for depicting small amounts of HPVG [7]. Wiesner et al. [8] reported that contrast-enhanced CT was a powerful investigatory tool to differentiate HPVG with acute mesenteric ischemia from non ischemic pathology.

Reports of intestinal pneumatosis have also been increasing [8–13]. Wiesner et al. [9] stated that band-like pneumatosis and the combination of pneumatosis and portomesenteric venous gas on CT are highly associated with transmural bowel infarction. DuBose et al. [10] conducted a retrospective multicenter study of 500 patients with pneumatosis intestinalis and reported that a lactate value of 2.0 or greater and hypotension/vasopressor use was associated with a predictive probability of 93.2 % of pathologic pneumatosis defined as confirmed transmural ischemia. Moreover, the reported specificities of pneumatosis and portal venous gas for acute bowel ischemia usually approach 100 % [8]. In contrast, according to some reports, intestinal pneumatosis is not useful for diagnosing the severity of HPVG [11]. Furthermore, neither pneumatosis nor portomesenteric venous gas is absolutely specific for transmural bowel wall necrosis in acute bowel ischemia, since the CT findings of both disorders may be observed in patients with only partial mural or even superficial mucosal and submucosal bowel ischemia, which are typically not associated with the same unfavorable clinical outcome [9]. The present study confirmed that intestinal pneumatosis is a significant independent risk factor for bowel necrosis.

Unexpected metabolic acidosis, as well as symptoms such as abdominal pain and peritoneal irritation, is indicative of mesenteric ischemia [6]. Another study suggested that increased lactate levels with anion gaps and/or CT findings suggestive of an ischemic bowel are indications for emergency laparotomy (“aggressive management”) [14]. Our findings are not in line with those of the aforementioned reports, which used different modalities to detect HPVG, evaluated a smaller sample size, comprised different articles (such as case reports and reviews), and did not perform a statistical analysis.

The acute physiology and chronic health evaluation (APACHE II) score is designed to measure severity of disease in adult patients admitted to intensive care units. Wu et al. [15] analyzed data for patients with ischemic bowel-induced HPVG and found that high APACHE II scores and longer length of bowel resection were associated with poor prognosis. To our knowledge, no reports have discussed the relationship between vital signs and bowel necrosis. Although some articles suggest that physical examinations are associated with bowel necrosis [6, 16], our findings did not show a significant correlation between physical examinations and bowel necrosis.

In this study, we created diagnostic criteria based on the three risk factors that were found to be significant independent factors for bowel necrosis. These factors have high sensitivity and accuracy, and can be evaluated easily by physicians in the emergency department. Nowadays, with the development of highly advanced imaging techniques, potentially severe pathologies, such as bowel ischemia, are diagnosed at much earlier stages, allowing prompt treatment and significantly lower mortality [17]. Although it is difficult to diagnose the cause of acute abdominal pain and bowel necrosis in patients with an unstable condition in the emergency department, our new criteria will allow physicians to establish the presence of bowel necrosis and perform surgery as quickly as possible.

The limitations of our study were that it was retrospective and the study population was small. Moreover, complete surgical or pathological and laboratory evaluations were not available for every patient. However, its findings warrant a study involving a larger sample size in the future. This study demonstrates new and significant findings related to the risk factors for bowel necrosis in patients with HPVG. Using our new diagnostic criteria, the indications for emergency laparotomy can be established more accurately.
